# Live birth rate per fresh embryo transfer and cumulative live birth rate in patients with PCOS under the POSEIDON classification: a retrospective study

**DOI:** 10.3389/fendo.2024.1348771

**Published:** 2024-05-28

**Authors:** Linlin Jiang, Yiting Sun, Ping Pan, Lin Li, Dongzi Yang, Jia Huang, Yu Li

**Affiliations:** ^1^ Department of Obstetrics and Gynecology, Reproductive Medicine Centre, Sun Yat-Sen Memorial Hospital, Sun Yat-Sen University, Guangzhou, China; ^2^ Department of Obstetrics and Gynecology, Guangdong Provincial Clinical Research Center for Obstetrical and Gynecological Diseases, Guangzhou, China; ^3^ The Assisted Reproductive Medicine Center, Northwest Women’s and Children’s Hospital, Xi’an, China; ^4^ Reproductive Medicine Centre, Shenshan Medical Center, Memorial Hospital of Sun Yat-Sen University, Shanwei, China

**Keywords:** polycystic ovarian syndrome, POSEIDON criteria, hypo-response, live birth rate, cumulative live birth rate

## Abstract

**Background:**

Ovarian stimulation (OS) for *in vitro* fertilization (IVF)/intracytoplasmic sperm injection (ICSI) in women with PCOS often results in multiple follicular development, yet some individuals experience poor or suboptimal responses. Limited data exist regarding the impact of poor/suboptimal ovarian response on pregnancy outcomes in women with PCOS.

**Objectives:**

The aim of this study was to evaluate whether the live birth rate (LBR) per fresh embryo transfer and cumulative live birth rate (CLBR) per aspiration cycle differ in women with PCOS defined by the Patient-Oriented Strategy Encompassing IndividualizeD Oocyte Number (POSEIDON) criteria.

**Methods:**

A retrospective study involving 2,377 women with PCOS who underwent their first IVF/ICSI cycle at Sun Yat-sen Memorial Hospital from January 2011 to December 2020 was used. Patients were categorized into four groups based on age, antral follicle count, and the number of oocytes retrieved, according to the POSEIDON criteria. The LBR and CLBR were compared among these groups. Logistic regression analysis was performed to assess whether the POSEIDON criteria served as independent risk factors and identify factors associated with POSEIDON.

**Results:**

For patients <35 years old, there was no significant difference in the clinical pregnancy rate between POSEIDON and non-POSEIDON patients, whereas POSEIDON patients exhibited lower rates of implantation and live birth. POSEIDON Group 1a displayed lower rates of implantation, clinical pregnancy, and live birth. However, no significant differences were observed in the rates of clinical pregnancy and live birth between POSEIDON Group 1b and non-POSEIDON groups. For patients ≥35 years old, there were no significant differences in the rates of implantation, clinical pregnancy, and live birth between POSEIDON and non-POSEIDON patients. CLBRs were significantly lower in POSEIDON Groups 1 and 2, compared with the non-POSEIDON groups. The levels of body mass index (BMI), follicle-stimulating hormone (FSH), and antral follicle count (AFC) were associated with POSEIDON hypo-response. POSEIDON was found to be associated with lower CLBR, but not LBR per fresh embryo transfer.

**Conclusions:**

In patients with PCOS, an unexpected suboptimal response can achieve a fair LBR per fresh embryo transfer. However, CLBR per aspirated cycle in POSEIDON patients was lower than that of normal responders. BMI, basal FSH level, and AFC were independent factors associated with POSEIDON. Our study provides data for decision-making in women with PCOS after an unexpected poor/suboptimal response to OS.

## Highlights

In patients with PCOS, an unexpected suboptimal response can achieve a fair LBR per fresh embryo transfer. However, CLBR per aspirated cycle in POSEIDON patients was lower than that of normal responders.

## Introduction

Polycystic ovarian syndrome (PCOS) is a heterogeneous endocrine disorder, which is characterized by ovulatory dysfunction, hyperandrogenism, and polycystic ovarian morphology (PCOM). Various factors including genetic, epigenetic, and environmental factors can influence PCOS (1–3). The prevalence of infertility in women with PCOS varies between 70% and 80% (4). *In vitro* fertilization (IVF) is the third-line treatment for infertility of women with PCOS (5). Challenges to IVF treatment in patients with PCOS include ovarian hyperstimulation syndrome (OHSS), impaired endothelial tolerance (6), altered oocyte competence (7), and increased risk of adverse pregnancy outcomes (8, 9). Adoption of an optimal ovarian stimulation (OS) protocol to overcome OHSS challenges is highly important (10–12). The starting dose of gonadotropins (Gn) is often calculated individually based on the patient’s age, body mass index (BMI), and ovarian reserve (13). Previous studies have shown that the heterogeneity of PCOS disease affects ovarian response to Gn stimulation. Hyperandrogenism or excessively high anti-Mullerian hormone (AMH) require higher Gn doses (14, 15). These make it difficult to standardize optimal OS regimens. Patients and physicians encounter decision-making challenges when unexpected poor/suboptimal ovarian response is detected. This decreases the patient’s expectation of success and increases the risk of cancelled oocyte retrieval and repeated IVF treatment.

The Patient-Oriented Strategy Encompassing IndividualizeD Oocyte Number (POSEIDON) criteria have refined the concept of poor ovarian response (POR) and allowed for the identification of infertile women with a low probability of pregnancy during IVF (16). Using this classification system, patients are grouped into four categories according to their age, ovarian reserve [antral follicle count (AFC) and/or AMH], and the number of oocytes retrieved from previous standard ovarian stimulation cycles: Group 1 includes patients who are under 35 years of age with adequate ovarian reserve; Group 2 comprises patients who are 35 years or older with adequate ovarian reserve; Group 3 consists of patients under 35 years with poor ovarian reserve; Group 4 includes patients who are 35 years or older with poor ovarian reserve (16).

Not all women with PCOS are high or normal responders to OS. Although not a high proportion, some women with PCOS fulfill the POSEIDON group, particularly for Groups 1 and 2. However, there are limited data available regarding the pregnancy outcomes of women with PCOS classified as poor responders (17, 18). There is no study to address LBR per fresh embryo transfer and CLBR per aspiration cycle for women with PCOS categorized as poor responders in fertility treatments.

This study aimed to assess LBR per fresh embryo transfer and CLBR per aspiration cycle, in women with PCOS categorized under POSEIDON and non-POSEIDON groups. Additionally, we wanted to investigate the potential variables associated with the occurrence of POSEIDON.

## Materials and methods

### Study population

This is a single-center retrospective study, which analyzes the records of women with PCOS who received IVF/intracytoplasmic sperm injection (ICSI) treatment at Sun Yat-sen Memorial Hospital. The study included patients aged between 20 and 46 years old, who underwent first cycle from January 2011 to December 2020. Inclusion criteria were (i) PCOS diagnosis according to the Rotterdam criteria; (ii) treatment with a standard ovarian stimulation protocol [GnRH antagonist (GnRH-ant) protocol or long GnRH agonist (GnRH-a) protocol]; and (iii) oocyte retrieval and delivery of a live birth after fresh or frozen–thawed embryo transfer (ET/FET), or no live birth delivery after transferring all embryos (19). Exclusion criteria were patients undergoing preimplantation genetic testing, those having ovarian operation history, or those who were lost to follow-up. The study was approved by the Sun Yat-sen Memorial Hospital ethical review board (SYSKY-2023–200-02). The anonymity of the data waived the need for informed consent.

### Clinical procedures

All participants received treatment with standard ovarian stimulation protocol using either the GnRH-a or the GnRH-ant protocol. Individualized doses of recombinant FSH or highly purified FSH were used based on ovarian reserve, age, and body weight, then doses were adjusted according to serum estradiol levels and follicular development. Oocyte maturation was induced via the administration of hCG, GnRH-a, or dual triggers. The retrieved oocytes were subjected to IVF or ICSI, and cultured reaching either Day 3 or the blastocyst stage. Embryo transfer was performed under the guidance of ultrasound and the remaining embryos were vitrified. Patients at risk of OHSS, with a premature progesterone elevation (i.e. >1.5 ng/mL), with endometrial polyps, and with a thin endometrium or intrauterine fluid used an embryo freezing strategy. Frozen embryo transfer was conducted in a natural cycle, an ovulation induction cycle, or a hormone replacement treatment cycle. Clinical pregnancy was confirmed by ultrasound. Luteal phase support was continued until 10–12 weeks of gestation for pregnant patients. Live birth delivery data were obtained by telephone interview.

### Patient classification

Women with PCOS were classified into four groups, retrospectively according to the POSEIDON criteria as described below. AFC was used as the biomarker for classification since it was available in all patients, whereas AMH levels were only available in 60.29% of cases (*n* = 1,433). In this study, AFC ranged from 12 to 80, hence, all women with PCOS exhibited either high or normal ovarian reserve, implying that no POSEIDON Group 3 or POSEIDON Group 4 patients were identified. Patients who did not meet the POSEIDON criteria were categorized as a separate group, termed “non-POSEIDON”. The classification criteria were as follows:

- POSEIDON Group 1 (Group 1): Comprising patients aged <35 years with AFC ≥5. Subgroup 1a included patients with <4 oocytes retrieved, and Subgroup 1b included patients with 4–9 oocytes retrieved.
- POSEIDON Group 2 (Group 2): Comprising patients aged ≥35 years with AFC ≥5. Subgroup 2a included patients with <4 oocytes retrieved, and Subgroup 2b included patients with 4–9 oocytes retrieved.
- Non-POSEIDON (Groups 3 and Group 4): Comprising patients with AFC ≥5 and >9 oocytes retrieved. Group 3 included patients aged <35 years old, and Group 4 included patients ≥35 years old.

### Main outcome measures

The main aim of this study was to determine the LBR per fresh embryo transfer. The CLBR per aspiration cycle with at least one live birth was also calculated. The criteria for live birth were defined according to the guidelines established by the International Committee for Monitoring Assisted Reproductive Technologies (ICMART) (19). A single or multiple delivery was recorded as one delivery.

### Statistical analysis

Continuous data were tested using the Shapiro–Wilk method. All the continuous data were non-normally distribution and presented as median and 25%–75% interquartile range, which were analyzed using the Mann-Whitney *U*-test. Categorical data were described by the percentages and were analyzed using Pearson chi-square. A *p*-value of <0.05 was considered as a statistically significant difference. Logistic regression analysis was performed to identify clinically significant variables associated with POSEIDON, and to assess whether the POSEIDON criteria served as independent risk factors for affecting LBR and CLBR. The results were reported as odds ratio (OR) with 95% confidence interval (CI). Patient covariates did not include AMH due to a high frequency of missing values. Data analysis was performed using IBM SPSS Statistics for Windows, Version 24.0 (IBM Corp., Armonk, NY, USA).

## Results

This study included a total of 2,377 patients with PCOS, of whom 547 patients met the POSEIDON criteria. The POSEIDON patients were stratified into one of the low-prognosis groups: Group 1a (*n* = 44), Group 1b (*n* = 430), Group 2a (*n* = 6), and Group 2b (*n* = 67). Non-POSEIDON patients were further divided into two groups based on age: <35 years old (*n* = 1,618) and ≥35 years old (*n* = 212) (**Supplementary Figure 1**). **Supplementary Figure 2** displays the distribution of patients in different groups across years. Patient characteristics, treatment parameters, fertilization rate, embryo development, and pregnancy outcomes were compared between the POSEIDON and non-POSEIDON groups within the same age stratum.

### Baseline information of patients

The baseline characteristics of women with PCOS included in our study are displayed in **Table 1**. Among patients <35 years old, no significant difference was found in age, infertility type, duration of infertility duration, and basal LH level between POSEIDON Group 1 and the non-POSEIDON groups. However, POSEIDON Group 1 had a higher BMI and basal FSH level, as well as lower AMH and AFC compared to the non-POSEIDON groups. Among patients ≥35 years old, there were no significant differences in baseline characteristics between POSEIDON Group 2 and the non-POSEIDON groups.

**Table 1:** Baseline characteristics of women with PCOS stratified according to the POSEIDON criteria and age.

		<35 years old					≥35 years old				
		POSEIDON			Non-POSEIDON	*p*1	POSEIDON			Non-POSEIDON	*p*2
		1a (*n* = 44)	1b (*n* = 430)	1 (*n* = 474)	3 (*n* = 1618)		2a (*n* = 6)	2b (*n* = 67)	2 (*n* = 73)	4 (*n* = 212)	
**Female age (years)**		29 (26, 31)	30 (27, 31)	29 (27, 31)	29 (27, 31)	0.086	37 (36, 37)	36 (35, 38)	36 (35, 38)	36 (35, 37)	0.728
**BMI (kg/m^2^)**		23 (21, 26)	22 (20, 25)	22 (20, 25)	21.5 (19.5, 23.5)	<0.001	23 (22, 26)	24 (21, 27)	23.8 (21.5, 26.7)	23.6 (21.1, 25.9)	0.223
Infertility type % (*n*)											
	**Primary**	65.9 (29/44)	61.2 (263/430)	61.6 (292/474)	60.3 (976/1,619)	0.631	33.3 (2/6)	47.8 (32/67)	46.6 (34/73)	38.7 (82/212)	0.270
	**Secondary**	34.1 (15/44)	38.8 (167/430)	38.4 (182/478)	39.7 (643/1,619)		66.7 (4/6)	52.2 (35/67)	53.4 (39/73)	61.3 (130/212)	
**Infertility duration (years)**		4 (2, 6)	4 (2, 6)	4 (2,6)	4 (2, 5)	0.064	5 (2, 9)	6 (3, 8)	6 (3,9)	5 (3, 8)	0.813
**Basal FSH (U/L)**		7.1 (6.0, 8.4)	7.1 (6.1, 8.2)	7.1 (6.1, 8.1)	6.8 (5.8, 9.1)	<0.001	6.7 (5.4, 7.4)	6.7 (5.7, 7.8)	6.8 (5.7, 7.6)	6.7 (5.7, 7.5)	0.510
**Basal LH (U/L)**		7.0 (3.9, 10.5)	6.5 (4.5, 10.0)	6.6 (4.4, 9.9)	6 (4.2, 9.6)	0.769	6.0 (4.5, 13.8)	6.5 (4.0, 9.0)	6.0 (3.8, 9.8)	5.8 (4.1, 9.2)	0.788
**AFC (*n*)**		28 (24, 35)	27 (24, 33)	28 (24, 33)	29 (24, 35)	0.008	25 (24, 45)	27 (24, 31)	26 (24, 30)	28 (24, 33)	0.157
**AMH (ng/mL)^#^**		7.5 (4.9, 9.4)	8.0 (5.8, 11.5)	7.8 (5.8, 11)	9.2 (6.2, 12.7)	0.001	11.4 (2.3, 17.8)	6.1 (4.0, 8.9)	6.1 (4.0,9.2)	7.2 (5.1, 10.0)	0.086

Values are median and interquartile range and percentage. BMI, body mass index; FSH, follicle-stimulating hormone; LH, luteinizing hormone; AFC, antral follicle count; AMH, anti-Müllerian hormone. p1, p-value between Group 1 (combined 1a+1b) and younger non-POSEIDON; p2, p-value between Group 2 (combined 2a+2b) and older non-POSEIDON. ^#^ based on data of 1,312 patients.

### Treatment characteristics

**Table 2** presents the treatment characteristics. The initial Gn dose was similar between POSEIDON and non-POSEIDON groups. However, POSEIDON Group 1 had longer stimulation duration and higher total Gn dose compared to the non-POSEIDON groups. The proportion of ovarian stimulation protocols, including GnRH-a and GnRH-ant, was similar between the POSEIDON and non-POSEIDON groups.

**Table 2:** Treatment characteristics of women with PCOS stratified according to the POSEIDON criteria and age.

	<35 years old					≥35 years old				
	POSEIDON			Non-POSEIDON	*p*1	POSEIDON			Non-POSEIDON	*p*2
	1a (*n* = 44)	1b (*n* = 430)	1 (*n* = 474)	3 (*n* = 1618)		2a (*n* = 6)	2b (*n* = 67)	2 (*n* = 73)	4 (*n* = 212)	
**Total Gn dose (IU)**	1,706 (1,303, 2,381)	1,588 (1,200, 2,100)	1,600 (1,200, 2,100)	1,350 (1,100, 1,763)	<0.001	1,781 (1,462, 2,475)	1,800 (1,431, 2,438)	1,800 (1,463, 2,400)	1,700 (1,350, 2,400)	0.291
**Initial Gn dose (IU)**	113 (112, 150)	113 (100, 150)	112.5 (100, 150)	113 (100, 150)	0.990	150 (112, 175)	150 (112, 150)	150 (113, 150)	150 (113, 150)	0.642
**Stimulation duration (days)**	13 (10, 15)	11 (9, 13)	11 (9, 13)	10 (9, 12)	<0.001	11 (9, 15)	11 (9, 14)	11 (9, 14)	11 (9, 12)	0.176
**Fertilization mode % (*n*)**					0.021					0.865
IVF	81.8 (36/44)	74.9 (322/430)	75.5 (358/474)	69.8 (1,129/1,618)		66.7 (4/6)	71.6 (48/67)	71.2 (52/73)	70.3 (149/212)	
ICSI	18.2 (8/44)	11.6 (50/430)	12.2 (58/474)	13.1 (212/1,618)		33.3 (2/6)	10.4 (7/67)	12.3 (9/73)	10.8 (23/212)	
Half-ICSI	0	13.5 (58/430)	12.2 (58/474)	17.1 (277/1,618)		0	17.9 (12/67)	16.4 (12/73)	18.9 (40/212)	
Protocol % (*n*)										
GnRH-a	43.2 (19/44)	44.4 (191/430)	44.3 (210/474)	49.1 (795/1,618)	0.064	33.3 (2/6)	50.7 (34/67)	49.3 (36/73)	59.0 (125/212)	0.172
GnRH-ant	56.8 (25/44)	55.6 (239/430)	55.7 (264/474)	50.9 (823/1,618)		66.7 (4/6)	49.3 (33/67)	50.7 (37/73)	41.0 (87/212)	

Values are median and interquartile range and percentage. BMI, body mass index; FSH, follicle-stimulating hormone; LH, luteinizing hormone; AFC, antral follicle count; AMH, anti-Müllerian hormone. p1, p-value between Group 1 (combined 1a+1b) and younger non-POSEIDON; p2, p-value between Group 2 (combined 2a+2b) and older non-POSEIDON.

### Fertilization rate and embryo development

Fertilization rate and embryo development are presented in **Table 3**. The POSEIDON patients had an over 2-fold lower number of oocytes retrieved than their non-POSEIDON counterparts. The number of usable embryo and top embryo was lower in the POSEIDON groups than in the non-POSEIDON groups.

**Table 3:** Fertilization rate and embryo development of women with PCOS stratified according to the POSEIDON criteria and age.

	<35 years old					≥35 years old				
	POSEIDON			Non-POSEIDON	*p*1	POSEIDON			Non-POSEIDON	*p*2
	1a (*n* = 44)	1b (*n* = 430)	1 (*n* = 474)	3 (*n* = 1618)		2a (*n* = 6)	2b (*n* = 67)	2 (*n* = 73)	4 (*n* = 212)	
Oocytes retrieved (*n*)	2.5 (2, 3)	7 (6, 9)	7 (5, 8)	17 (13, 22)	<0.001	2 (1.75, 3)	7 (6, 9)	7 (5, 8)	16 (13, 20)	<0.001
2PN zygote rate % (*n*)	88.3 (76/86)*	81.4 (1,942/2,386)*	81.6 (2,018/2,472)	78.9 (17,609/22,312)	0.002	80.0 (8/10)	73.2 (262/358)*	73.4 (270/368)	81.2 (2,230/2,747)	0.001
Useable embryo (*n*)	2 (1, 2)	3 (2, 5)	3 (2, 5)	7 (4, 10)	<0.001	1.5 (0, 2.25)	3 (2, 4)	3 (2, 4)	7 (4, 9)	<0.001
Useable embryo rate % (*n*)	88.2 (67/76)*	81.6 (1,585/1,942)*	81.9 (1,652/2,018)	59.5 (12,699/21,352)	<0.001	100 (8/8)	85.5 (224/262)*	85.9 (232/270)	67.4 (1,502/2,230)	<0.001
Top embryo (*n*)	0 (0, 1)	1 (0, 2)	1 (0, 2)	2 (0, 5)	<0.001	0.5 (0, 1.25)	1 (0, 1.5)	1 (0, 1)	2 (0, 4)	<0.001
Top embryo rate % (*n*)	41.8 (28/67)	42.7 (677/1,585)*	42.7 (705/1,652)	47.6 (6,040/12,696)	<0.001	62.5 (5/8)	38.4 (86/224)*	39.2 (91/232)	47.3 (711/1,502)	0.023

p1, p-value between Group 1 (combined 1a+1b) and younger non-POSEIDON; p2, p-value between Group 2 (combined 2a+2b) and older non-POSEIDON. *Compared with its non-POSEIDON counterpart in same age stratum and p<0.05.

### Pregnancy outcomes

Pregnancy outcomes are displayed in **Table 4**. The rate of fresh embryo transfer cancellation was significantly higher and more frozen embryo transfers were conducted in the non-POSEIDON groups compared with their POSEIDON counterparts. For patients <35 years old, the rates of implantation and live birth per fresh transfer were significantly lower in POSEIDON patients, whereas the clinical pregnancy rate had no significant difference. **Supplementary Table 1** displays the pregnancy outcomes for Group 1a and Group 1b, which showed no significant differences of clinical pregnancy rate and live birth rate between POSEIDON Group 1b and the non-POSEIDON groups (60.1% vs. 63.2%, *p* = 0.276; 50.0% vs. 55.4%, *p* = 0.070). For patients ≥35 years old, there were no significant differences in the rates of implantation, clinical pregnancy, and live birth per fresh transfer between POSEIDON and non-POSEIDON patients, as well as Group 2a and Group 2b (**Supplementary Table 2**). For FET cycles, LBR per FET was significantly lower in Group 1 compared with the non-POSEIDON groups, but Group 2 did not reach statistical difference. The CLBRs in both age groups were lower in the POSEIDON groups compared to the non-POSEIDON groups (<35 years old: 56.5% vs. 81.0%, *p* < 0.001; ≥35 years old: 50.7% vs. 74.1%, *p* < 0.001). POSEIDON suboptimal responders (Group 1b: 58.6%; Group 2b: 52.2%) had higher CLBR than poor responders (Group 1a: 36.4%; Group 2a: 33.3%). Fresh embryo transfer was the most common conception mode to achieve a live birth delivery for POSEIDON patients in both age groups (75.7% for Group 1, 91.9% for Group 2).

**Table 4:** Pregnancy outcomes of women with PCOS stratified according to the POSEIDON criteria.

	<35 years old					≥35 years old				
	POSEIDON			Non-POSEIDON	*p*1	POSEIDON			Non-POSEIDON	*p*2
	1a (*n* = 44)	1b (*n* = 430)	1 (*n* = 474)	3 (*n* = 1618)		2a (*n* = 6)	2b (*n* = 67)	2 (*n* = 73)	4 (*n* = 212)	
	1a (*n* = 44)	1b (*n* = 430)	1 (*n* = 474)	3 (*n* = 1618)		2a (*n* = 6)	2b (*n* = 67)	2 (*n* = 73)	4 (*n* = 212)	
ET cancel rate % (*n*)	15.9 (7/44)*	12.1 (52/430)*	12.4 (50/474)	38.1 (617/1,618)	<0.001	33.3 (2/6)	4.5 (3/67)*	6.7 (5/75)	35.8 (76/212)	<0.001
Type of transfer										
Fresh	77.3 (34/44)	63.7 (274/430)	65.0 (308/474)	37.5 (607/1,618)	<0.001	50 (3/6)	77.6 (52/67)	75.3 (55/73)	42.0 (89/212)	<0.001
FET	2.3 (1/44)	10.2 (44/430)	9.5 (45/474)	39.5 (639/1,618)		0	3.0 (2/67)	2.7 (2/73)	34.9 (74/212)	
Fresh + FET	6.8 (3/44)	24.2 (104/430)	22.6 (107/474)	22.4 (362/1,618)		16.7 (1/6)	17.9 (12/67)	17.8 (13/73)	22.2 (47/212)	
No transfer	13.6 (6/44)	1.9 (8/430)	3.0 (14/474)	0.6 (10/1,618)		33.3 (2/6)	1.5 (1/67)	4.1 (3/73)	0.9 (2/212)	
Fresh cycles (*n* = 1,620)	*n* = 37	*n* = 378	*n* = 415	*n* = 1001		*n* = 4	*n* = 64	*n* = 68	*n* = 136	
Embryo transferred (*n*)	2 (1,2)*	2 (2,2)*	2 (2,2)	2 (0,2)	0.682	1 (0,1.3)*	2 (2,3)*	2 (2,2)	2 (0,2)	0.242
Blastocyte transferred (%)	0*	2.1 (8/378)*	1.9 (8/415)	10.5 (105/1,001)	<0.001	0	6.3 (4/64)*	5.9 (4/68)	15.4 (21/136)	0.068
Implantation rate % (*n*)	33.9 (21/62)*	42.8 (307/717)*	42.1 (239/411)	47.6 (879/1,847)	0.01	20.0 (1/5)	37.3 (53/142)	36.7 (54/147)	43.7 (124/284)	0.181
Clinical pregnancy rate % (*n*)	43.2 (16/37)*	60.1 (227/378)	58.6 (243/415)	63.2 (633/1,001)	0.105	25.0 (1/4)	62.5 (40/64)	60.3 (41/68)	67.6 (92/136)	0.35
LBR per transfer fresh % (*n*)	37.8 (14/37)*	50.0 (189/378)	48.9 (203/415)	55.4 (555/1,001)	0.026	25.0 (1/4)	51.6 (33/64)	40.0 (34/68)	57.4 (78/136)	0.371
FET cycles (*n* = 1,898)	*n* = 4	*n* = 188	*n* = 192	*n* = 1490		*n* = 1	*n* = 15	*n* = 16	*n* = 200	
LBR per transfer FET % (*n*)	50.0 (2/4)	33.5 (63/188)*	33.9 (65/192)	50.7 (755/1,490)	<0.001	100.0 (1/1)	13.3 (2/15)*	20.0 (3/15)	39.5 (79/200)	0.115
CLBR % (*n*)	36.4 (16/44)*	58.6 (252/430)*	56.5 (268/474)	81.0 (1,310/1,618)	<0.001	33.3 (2/6)	52.2 (35/67)*	50.7 (37/73)	74.1 (157/212)	<0.001
Conception mode % (*n*)										
IVF/ICSI fresh % (*n*)	87.5 (14/16)*	75.0 (189/252)*	75.7 (203/268)	42.4 (555/1,310)	<0.001	50 (1/2)	94.3 (33/35)*	91.9 (34/37)	49.7 (78/157)	<0.001
IVF/ICSI FET % (*n*)	12.5 (2/16)	25.0 (63/252)	24.3 (65/268)	57.6 (755/1,310)		50 (1/2)	5.7 (2/35)	8.1 (3/37)	50.3 (79/157)	

CLBR, cumulative live birth rate: cumulative delivery rate from one aspiration IVF/ICSI cycle. p1, p-value between Group 1 (combined 1a+1b) and younger non-POSEIDON; p2, p-value between Group 2 (combined 2a+2b) and older non-POSEIDON. *Compared with its non-POSEIDON counterpart in same age stratum and p<0.05.

### Logistic regression analysis

**Tables 5**–**7** showed the OR and adjusted OR (aOR) with their 95% CI for POSEIDON hypo-response, LBR per fresh embryo transfer, and CLBR per oocyte aspiration in patients with PCOS. BMI, basal FSH level, and AFC were significantly associated with POSEIDON hypo-response after adjusting for confounders (BMI: aOR 1.085, *p* < 0.001; basal FSH: aOR 1.095, *p* = 0.001; and AFC: aOR 0.988, *p* = 0.033). As for LBR, POSEIDON Group 1a was associated with a significantly lower LBR, even after adjusting for important confounders such as age, BMI, and the type of infertility. However, POSEIDON Group 1b, POSEIDON Group 2a, and POSEIDON Group 2b had no significant association with LBR. As for CLBR, both POSEIDON Group 1 and POSEIDON Group 2 were significantly associated with lower CLBR even after adjusting for important confounders (Group 1: OR 0.308, 95% CI 0.246–0.384, *p* < 0.001; and Group 2: OR 0.337, 95% CI 0.188–0.606, *p* < 0.001).

**Table 5:** Odds ratio for POSEIDON hypo-response of women with PCOS.

Indicators	OR (95% CI)	*p*-value	Adjusted OR (95% CI)	Adjusted *p*-value
**Age (years)**	1.023 (0.998, 1.050)	0.076	1.009 (0.983, 1.035)	0.518
**BMI (kg/m^2^)**	1.079 (1.049, 1.111)	<0.001	1.085 (1.053, 1.118)	<0.001
Infertility type				
**Primary**	1.076 (0.886, 1.307)	0.458	–	–
**Secondary**	1.000			
**Basal FSH (U/L)**	1.078 (1.025, 1.134)	0.004	1.095 (1.039, 1.153)	0.001
**Basal LH (U/L)**	0.994 (0.977, 1.011)	0601	–	–
**AFC (*n*)**	0.985 (0.974, 0.996)	0.009	0.988 (0.976, 0.999)	0.033

OR, odds ratio.

**Table 6:** Odds ratio for LBR from one aspiration IVF/ICSI cycle according to POSEIDON groups.

	Patient category	Unadjusted OR (95% CI)	*p*-value	Adjusted OR (95% CI)	*p*-value
**<35 years old**	**Non-POSEIDON**	**Reference**	–	–	–
	POSEIDON Group 1a	0.494 (0.249, 0.981)	0.044	0.491 (0.246, 0.977)	0.043
	POSEIDON Group 1b	0.834 (0.659, 1.057)	0.133	0.829 (0.654, 1.050)	0.120
	POSEIDON Group 1 (a + b combined)	0.798 (0.635, 1.003)	0.053	0.793 (0.630, 0.998)	0.048
**≥35 years old**	**Non-POSEIDON**	**Reference**	–	–	–
	POSEIDON Group 2a	0.296 (0.030, 2.920)	0.297	0.278 (0.028, 2.815)	0.279
	POSEIDON Group 2b	0.616 (0.339, 1.116)	0.110	0.581 (0.310, 1.089)	0.090
	POSEIDON Group 2 (a + b combined)	0.593 (0.330, 1.064)	0.080	0.558 (0.301, 1.035)	0.064

Age, BMI, and infertility type were adjusted in the multivariate analysis. OR, odds ratio.

**Table 7:** Odds ratio for CLBR from one aspiration IVF/ICSI cycle according to POSEIDON groups.

	Patient category	Unadjusted OR (95% CI)	*p*-value	Adjusted OR (95% CI)	*p-*value
**<35 years old**	**Non-POSEIDON**	**Reference**	–	–	–
	POSEIDON Group 1a	0.134 (0.072, 0.251)	<0.001	0.136 (0.072, 0.256)	<0.001
	POSEIDON Group 1b	0.333 (0.265, 0.418)	<0.001	0.333 (0.264, 0.420)	<0.001
	POSEIDON Group 1 (a + b combined)	0.306 (0.245, 0.381)	<0.001	0.308 (0.246, 0.384)	<0.001
**≥35 years old**	**Non-POSEIDON**	**Reference**	–	–	–
	POSEIDON Group 2a	0.175 (0.031, 0.983)	0.048	0.155 (0.027, 0.896)	0.035
	POSEIDON Group 2b	0.383 (0.217, 0.677)	0.001	0.365 (0.199, 0.669)	<0.001
	POSEIDON Group 2 (a + b combined)	0.360 (0.207, 0.625)	<0.001	0.337 (0.188, 0.606)	<0.001

Age, BMI, and infertility type were adjusted in the multivariate analysis. OR, odds ratio.

## Discussion

Ovarian stimulation is an important first step in IVF treatments. OS aims to obtain a sufficient number of oocytes in one cycle to maximize the patient’s pregnancy outcome. When considering the fresh embryo transfer LBR, optimal outcomes are achieved when retrieving between 6 and 15 oocytes (20, 21). When considering the cumulative LBR, studies have generally reported a positive trend where more oocytes result in a higher cumulative LBR (22, 23). However, when more oocytes are retrieved, the incidence of OHSS in the stimulation cycle increases considerably. Optimal OS strategies balance efficacy and safety. Following the ESHRE guidelines for OS, the Gn dose should not exceed 150 IU for high responders and should be initiated at a lower dose for those at risk (24). However, a low Gn dose leads to a risk of increased cancellation rate and decreased oocyte yield (25). In our study, starting doses below 150 IU accounted for 56% of cycles, and 23% had a poor or suboptimal response to OS. Among them, approximately 90% of patients exhibit a suboptimal response, with less than 10% of patients exhibiting a poor response. The mechanism why ovaries respond differently to Gn stimulation in women with PCOS is unclear. The heterogeneity of PCOS disease may be a possible explanation. The phenotype group (presence of both hyperandrogenism and chronic anovulation) tended to have higher doses of used Gn than the other PCOS phenotypes (14). Insulin resistance, a critical aspect of pathophysiology in patients with PCOS (26), may be associated with reduced ovarian sensitivity to exogenous Gn (27). More Gn were needed to obtain adequate oocytes in patients with PCOS with a higher BMI (28). High levels of AMH are highly correlated with PCOM and are a high-risk factor for developing OHSS. However, it has also been found that excessive AMH causes the ovaries to be resistant to Gn stimulation, requiring a higher Gn dose (15, 29). It was hypothesized that high concentrations of AMH would inhibit the action of FSH and thus be detrimental to the folliculogenesis process. Genetic variants in Gn and receptors may be another reason for differences in ovarian sensitivity. Polymorphisms of FSHR, as well as the variant of the β subunit of LH, have been associated with sensitivity to Gn (30, 31). In our study, we found that BMI, basal FSH level, and AFC may be potential influencing factors for POR in patients with PCOS. Further research is needed on the causes, risk factors, and predictors of unintended POR after OS in women with PCOS.

The POSEIDON classification system is anticipated to improve counseling and management of low-prognosis patients undergoing ART. Based on our limited search, this is the first study to focus on pregnancy outcomes in women with PCOS who have a poor/suboptimal response to OS. This study revealed that patients with PCOS categorized under the POSEIDON suboptimal response (Groups 1b and 2b) exhibited no statistically significant differences in the LBR per fresh embryo transfer compared to the non-POSEIDON groups (50.0% vs. 55.4%, *p* = 0.079; 51.6% vs. 57.4%, *p* = 0.450). However, patients meeting the POSEIDON poor response (Group 1a) had lower LBR per fresh embryo transfer when compared with the non-POSEIDON groups (37.8% vs. 55.4%, *p* = 0.035). Given that Group 2a included only four cases for fresh embryo transfer, the results of the statistical test comparing LBR to the non-POSEIDON groups were less meaningful due to the large standard error caused by the small sample size (25% vs. 57.4%, *p* = 0.198). This indicated that unexpected suboptimal response could achieve fair LBR per fresh embryo transfer in women with PCOS. However, all the POSEIDON groups (Groups 1a, 1b, 2a, and 2b) had lower CLBRs per aspirated IVF/ICSI cycle than normal responders. The CLBRs varied across different POSEIDON subgroups, with rates of 36.4%, 58.6%, 33.3%, and 52.2% for Group 1a, Group 1b, Group 2a, and Group 2b, respectively.

In our study, we found that in patients with PCOS, there were no statistically significant differences in the LBR per fresh embryo transfer between POSEIDON Group 1b and Group 2b compared with the non-POSEIDON groups. This indicated that only the quantity of oocytes was decreased, and not the quality, in unexpected suboptimal responders in women with PCOS. Our findings were consistent with other studies. Chinta et al. reported no significant differences in the LBR per fresh embryo transfer between POSEIDON Groups 1 and 2 and the non-POSEIDON groups (32). A study of 2,455 cycles found that the LBR after fresh transfer was highest in patients with 6–15 oocytes retrieved and reduced in patients with 0–5 or >15 oocytes retrieved (20). For women with PCOS <35 years old, it was found that the proportion of live births per fresh embryo transfer was similar for those who had less than 6 oocytes retrieved, and those who had 7–16 oocytes retrieved (33). These findings suggested that fair LBR per fresh embryo transfer can still be achieved in unexpected suboptimal responders. Moreover, the lower embryo transfer cancellation rate in POSEIDON groups may result in a shorter time to live birth, providing valuable information for counseling patients with unexpected suboptimal ovarian response in PCOS.

Here, we also found that LBR per FET in POSEIDON Group 1 was significantly lower than the non-POSEIDON groups paired with age (33.9% vs. 50.8%, *p* < 0.001). LBR per FET in POSEIDON Group 2 was also lower than its counterparts, though there was no statistical difference (20.0% vs. 39.5%, *p* = 0.115). This observation is expected, given that fewer oocytes were picked up and fewer good embryos were obtained in POSEIDON patients. In the FET cycle, available embryos were transferred after being selected for fresh embryo transfer. Therefore, the optimal number of oocytes is a critical factor in maximum CLBR for women with PCOS. The CLBR was significantly lower in POSEIDON Groups 1 and 2 compared with the non-POSEIDON groups paired with age (56.5% vs. 81.0%, *p* < 0.001; 50.7% vs. 74.1%, *p* < 0.001), according to our study. A retrospective study that included 18,455 cycles also showed lower CLBR in POSEIDON Groups 1 and 2 compared to the non-POSEIDON patients, which is consistent with our finding in women with PCOS (34). A real-world study of 9,073 patients, including 212 patients from Group 1a, 1,785 patients from Group 1b, 293 patients from Group 2a, 1,275 patients from Group 2b, and 4,640 non-POSEIDON patients, revealed that CLBR differed across the POSEIDON groups (Group 1a: 27.8%, Group 1b: 47.8%, Group 2a:14%, and Group 2b: 30.5%) and was lower than the non-POSEIDON groups (50.6%) (35). In our study, the CLBRs were 36.4%, 58.6%, 33.3%, and 52.2% in Group 1a, Group 1b, Group 2a, and Group 2b, respectively. These rates seemed higher compared to other studies and could be explained by the high ovarian reserve of women with PCOS. The CLBR after including frozen embryo transfer cycles increased with oocyte number (20). Similarly, in women with PCOS, high CLBR can be obtained with 15 or more oocytes retrieved (33). However, a higher number of oocytes retrieved does not necessarily lead to a better prognosis for patients with PCOS, but a higher surplus embryo rate. A retrospective study in women with PCOS found that CLBR could reach up to 81.91% when 10–15 oocytes were retrieved, but when the number of oocytes were ≥16, a higher risk of OHSS was achieved but not a higher CLBR (36).

The management of POR in women with PCOS is varied: cancellation of the cycle, switching to intrauterine insemination (if the tubal obstruction is not present), dual stimulation, switching from IVF to IVM as a salvage strategy (37), or transvaginal ovarian drilling followed by controlled ovarian stimulation from the next day onwards (38). Our study provides new data that when the expected number of oocytes is more than four, the live birth rate of a fresh transfer is not affected. When fewer than four oocytes are obtained, the live birth rate decreases but is still cost-effective considering the time and economic costs of retrieving the oocytes again. Our study can be useful for the counseling decision-making process.

Our study was the first to investigate LBR per fresh embryo transfer and CLBR per aspiration IVF/ICSI cycle in women with PCOS under the POSEIDON classification. However, a limitation of our study was the small number of cases in POSEIDON Group 2a, an issue that happened for observational data, which were too low to draw any definitive conclusions. Additionally, we were unable to calculate CLBR after all frozen embryos were transplanted, because preserved embryos were common in most women with PCOS. Therefore, we calculated the CLBR per aspiration cycle with at least one live birth after embryo transfer or did not get a live birth delivery after transferring all embryos. Further studies are needed to compare CLBR of women with PCOS and women without PCOS who were identified with POSEIDON. Another limitation was the high frequency of missing values of AMH for analysis. We have not analyzed PCOS phenotypes, although only specific PCOS phenotypes may fall into POSEIDON criteria. We hope that, in the future, we will gather more comprehensive baseline data on women with PCOS and conduct analyses of LBR across different phenotypes. All IVF data were derived from a single center, whereas the pregnancies were followed in different centers. The risk of pregnancy complications is high in patients with PCOS, and specialized monitoring could influence the data. This potentially limits the broad applicability of our results. Therefore, our findings necessitate further validation in a large and diverse population.

In this study, we found that in patients with PCOS, the unexpected suboptimal response could achieve fair LBR per fresh embryo transfer; however, the CLBR per aspirated cycle in POSEIDON patients was lower than that of normal responders. BMI, basal FSH level, and AFC were identified as independent factors associated with unexpected poor/suboptimal response to standard ovarian stimulation, while POSEIDON classification was an independent risk factor for CLBR. Therefore, the unexpected suboptimal response could achieve fair LBR per fresh embryo transfer, which can be useful information for counseling patients with unexpected suboptimal ovarian response in PCOS. Efforts should be made to recognize POSEIDON risk factors and obtain appropriate oocyte retrieval number to maximize IVF/ICSI outcomes for women with PCOS.

## Data availability statement

The raw data supporting the conclusions of this article will be made available by the authors, without undue reservation.

## Ethics statement

The studies involving humans were approved by Sun Yat-sen Memorial Hospital ethical review board (SYSKY-2023-200-02). The studies were conducted in accordance with the local legislation and institutional requirements. The anonymity of the retrospective data waived the need for informed consent.

## Author contributions

LJ: Writing – original draft, Formal analysis. YS: Writing – original draft, Formal analysis. PP: Writing – review & editing, Data curation. LL: Writing – review & editing, Data curation. DY: Writing – review & editing, Data curation. JH: Writing – review & editing, Methodology, Conceptualization. YL: Writing – review & editing, Methodology, Funding acquisition, Conceptualization.

## Supplementary material

Supplementary Figure 1 Flow diagram showing total patient breakdown. POSEIDON Group 1 (Group 1): Age <35 years and AFC ≥5. Subgroup 1a included patients with fewer than four oocytes retrieved, and Subgroup 1b included patients with four to nine oocytes retrieved. POSEIDON Group 2 (Group 2): Age ≥35 years and AFC ≥5. Subgroup 2a included patients with fewer than four oocytes retrieved, and Subgroup 2b included patients with four to nine oocytes retrieved. Non-POSEIDON (Group 3 and Group 4): Patients with AFC ≥5 and >9 oocytes retrieved. Group 3 included patients aged <35 years, and Group 4 included patients aged 35 years and older.

Supplementary Figure 2 Bar chart showing the distribution of patients in each POSEIDON group in different years from 2011 to 2020.

## References

[1] Zhou G Gu Y Zhou F Zhang H Zhang M Zhang G. Adipocytes-derived extracellular vesicle-miR-26b promotes apoptosis of cumulus cells and induces polycystic ovary syndrome. Front Endocrinol (Lausanne). (2021) 12:789939. doi: 10.3389/fendo.2021.789939 35222263 PMC8873091

[2] Day F Karaderi T Jones MR Meun C He C Drong A. Large-scale genome-wide meta-analysis of polycystic ovary syndrome suggests shared genetic architecture for different diagnosis criteria. PloS Genet. (2018) 14:e1007813. doi: 10.1371/journal.pgen.1007813 30566500 PMC6300389

[3] Gu Y Zhou G Zhou F Li Y Wu Q He H. Gut and vaginal microbiomes in PCOS: implications for women’s health. Front Endocrinol (Lausanne). (2022) 13:808508. doi: 10.3389/fendo.2022.808508 35282446 PMC8905243

[4] Joham AE Teede HJ Ranasinha S Zoungas S Boyle J. Prevalence of infertility and use of fertility treatment in women with polycystic ovary syndrome: data from a large community-based cohort study. J Womens Health (Larchmt). (2015) 24:299–307. doi: 10.1089/jwh.2014.5000 25654626

[5] Teede HJ Tay CT Laven JJE Dokras A Moran LJ Piltonen TT. Recommendations from the 2023 international evidence-based guideline for the assessment and management of polycystic ovary syndrome. J Clin Endocrinol Metab. (2023) 108:2447–69. doi: 10.1210/clinem/dgad463 PMC10505534 37580314

[6] Palomba S Piltonen TT Giudice LC. Endometrial function in women with polycystic ovary syndrome: a comprehensive review. Hum Reprod Update. (2021) 27:584–618. doi: 10.1093/humupd/dmaa051 33302299

[7] Palomba S Daolio J La Sala GB. Oocyte competence in women with polycystic ovary syndrome. Trends Endocrinol Metab. (2017) 28:186–98. doi: 10.1016/j.tem.2016.11.008 27988256

[8] Palomba S. Is fertility reduced in ovulatory women with polycystic ovary syndrome? An opinion paper. Hum Reprod. (2021) 36:2421–8. doi: 10.1093/humrep/deab181 34333641

[9] Palomba S de Wilde MA Falbo A Koster MPH La Sala GB Fauser BCJM. Pregnancy complications in women with polycystic ovary syndrome. Hum Reprod Update. (2015) 21:575–92. doi: 10.1093/humupd/dmv029 26117684

[10] Palomba S Costanzi F Nelson SM Besharat A Caserta D Humaidan P. Beyond the umbrella: A systematic review of the interventions for the prevention of and reduction in the incidence and severity of ovarian hyperstimulation syndrome in patients who undergo *in vitro* fertilization treatments. Int J Mol Sci. (2023) 24:14185. doi: 10.3390/ijms241814185 37762488 PMC10531768

[11] Palomba S Costanzi F Nelson SM Caserta D Humaidan P. Interventions to prevent or reduce the incidence and severity of ovarian hyperstimulation syndrome: a systematic umbrella review of the best clinical evidence. Reprod Biol Endocrinol. (2023) 21:67. doi: 10.1186/s12958-023-01113-6 37480081 PMC10360244

[12] Sun B Ma Y Li L Hu L Wang F Zhang Y. Factors associated with ovarian hyperstimulation syndrome (OHSS) severity in women with polycystic ovary syndrome undergoing IVF/ICSI. Front Endocrinol. (2020) 11:615957. doi: 10.3389/fendo.2020.615957 PMC7851086 33542709

[13] Pinborg A Gaarslev C Hougaard CO Nyboe Andersen A Andersen PK Boivin J. Influence of female bodyweight on IVF outcome: a longitudinal multicentre cohort study of 487 infertile couples. Reprod BioMed Online. (2011) 23:490–9. doi: 10.1016/j.rbmo.2011.06.010 21856228

[14] Cela V Obino MER Alberga Y Pinelli S Sergiampietri C Casarosa E. Ovarian response to controlled ovarian stimulation in women with different polycystic ovary syndrome phenotypes. Gynecol Endocrinol. (2018) 34:518–23. doi: 10.1080/09513590.2017.1412429 29271274

[15] Kamel A Ramadan W Hussein AM Dahab S Elsherbini MM Lasheen YS. Can AMH levels predict the need for increased medication during IVF/ICSI in PCOS women? J Matern Fetal Neonatal Med. (2018) 31:32–8. doi: 10.1080/14767058.2016.1272567 27978775

[16] Esteves SC Roque M Bedoschi GM Conforti A Humaidan P Alviggi C. Defining low prognosis patients undergoing assisted reproductive technology: POSEIDON criteria-the why. Front Endocrinol (Lausanne). (2018) 9:461. doi: 10.3389/fendo.2018.00461 30174650 PMC6107695

[17] Yang X Jiang M Deng M Zhang H Lin Z Fei X. Clomiphene citrate mild stimulation improved follicular development outcomes in PCOS women with high luteinizing hormone and poor ovarian response: A case report. Med (Baltimore). (2022) 101:e31323. doi: 10.1097/MD.0000000000031323 PMC9592273 36281179

[18] Burnik Papler T Stimpfel M Kovacik B Bokal EV. Poor ovarian response to gonadotrophins in PCOS women after laparoscopic ovarian drilling. Medicina (Kaunas). (2022) 58:147. doi: 10.3390/medicina58020147 35208471 PMC8879148

[19] Zegers-Hochschild F Adamson GD Dyer S Racowsky C de Mouzon J Sokol R. The international glossary on infertility and fertility care, 2017. Hum Reprod. (2017) 32:1786–801. doi: 10.1093/humrep/dex234 PMC5850297 29117321

[20] Ji J Liu Y Tong XH Luo L Ma J Chen Z. The optimum number of oocytes in IVF treatment: an analysis of 2455 cycles in China. Hum Reprod. (2013) 28:2728–34. doi: 10.1093/humrep/det303 23887075

[21] Magnusson Å Källen K Thurin-Kjellberg A Bergh C. The number of oocytes retrieved during IVF: a balance between efficacy and safety. Hum Reprod. (2018) 33:58–64. doi: 10.1093/humrep/dex334 29136154

[22] Fanton M Cho JH Baker VL Loewke K. A higher number of oocytes retrieved is associated with an increase in fertilized oocytes, blastocysts, and cumulative live birth rates. Fertil Steril. (2023) 119:762–9. doi: 10.1016/j.fertnstert.2023.01.001 36634732

[23] Polyzos NP Drakopoulos P Parra J Pellicer A Santos-Ribeiro S Tournaye H. Cumulative live birth rates according to the number of oocytes retrieved after the first ovarian stimulation for *in vitro* fertilization/intracytoplasmic sperm injection: a multicenter multinational analysis including ∼15,000 women. Fertil Steril. (2018) 110:661–670.e1. doi: 10.1016/j.fertnstert.2018.04.039 30196963

[24] Ovarian Stimulation TEGGO Bosch E Broer S Griesinger G Grynberg M Humaidan P. ESHRE guideline: ovarian stimulation for IVF/ICSI(†). Hum Reprod Open. (2020) 2020:hoaa009. doi: 10.1093/hropen/hoaa009 32395637 PMC7203749

[25] Oudshoorn SC van Tilborg TC Eijkemans MJC Oosterhuis GJE Friederich J van Hooff MHA. Individualized versus standard FSH dosing in women starting IVF/ICSI: an RCT. Part 2: The predicted hyper responder. Hum Reprod. (2017) 32:2506–14. doi: 10.1093/humrep/dex319 29121269

[26] Gu Y Zhou G Zhou F Wu Q Ma C Zhang Y. Life modifications and PCOS: old story but new tales. Front Endocrinol (Lausanne). (2022) 13:808898. doi: 10.3389/fendo.2022.808898 35498415 PMC9045543

[27] Li Y Wang Y Liu H Zhang S Zhang C. Association between HOMA-IR and ovarian sensitivity index in women with PCOS undergoing ART: A retrospective cohort study. Front Endocrinol (Lausanne). (2023) 14:1117996. doi: 10.3389/fendo.2023.1117996 36967765 PMC10034104

[28] McCormick B Thomas M Maxwell R Williams D Aubuchon M. Effects of polycystic ovarian syndrome on *in vitro* fertilization-embryo transfer outcomes are influenced by body mass index. Fertil Steril. (2008) 90:2304–9. doi: 10.1016/j.fertnstert.2007.10.077 18191852

[29] Huang H Gao H Shi Y Deng B He X Lin J. Can AMH levels predict the need to step up FSH dose for controlled ovarian stimulation following a long GnRH agonist protocol in PCOS women? Reprod Biol Endocrinol. (2023) 21:121. doi: 10.1186/s12958-023-01173-8 38110998 PMC10726541

[30] Alviggi C Pettersson K Longobardi S Andersen CY Conforti A De Rosa P. A common polymorphic allele of the LH beta-subunit gene is associated with higher exogenous FSH consumption during controlled ovarian stimulation for assisted reproductive technology. Reprod Biol Endocrinol. (2013) 11:51. doi: 10.1186/1477-7827-11-51 23725475 PMC3694457

[31] Yao Y Ma CH Tang HL Hu YF. Influence of follicle-stimulating hormone receptor (FSHR) Ser680Asn polymorphism on ovarian function and *in-vitro* fertilization outcome: a meta-analysis. Mol Genet Metab. (2011) 103:388–93. doi: 10.1016/j.ymgme.2011.04.005 21546300

[32] Chinta P Antonisamy B Mangalaraj AM Kunjummen AT Kamath MS. POSEIDON classification and the proposed treatment options for groups 1 and 2: time to revisit? A retrospective analysis of 1425 ART cycles. Hum Reprod Open. (2021) 2021:hoaa070. doi: 10.1093/hropen/hoaa070 33614989 PMC7882041

[33] Jia R Liu Y Jiang R Zhu X Zhou L Chen P. The optimal number of oocytes retrieved from PCOS patients receiving IVF to obtain associated with maximum cumulative live birth rate and live birth after fresh embryo transfer. Front Endocrinol (Lausanne). (2022) 13:878214. doi: 10.3389/fendo.2022.878214 35813639 PMC9259927

[34] Shi W Zhou H Tian L Zhao Z Zhang W Shi J. Cumulative live birth rates of good and low prognosis patients according to POSEIDON criteria: A single center analysis of 18,455 treatment cycles. Front Endocrinol (Lausanne). (2019) 10:409. doi: 10.3389/fendo.2019.00409 31293519 PMC6606694

[35] Esteves SC Yarali H Vuong LN Carvalho JF Ozbek IY Polat M. Cumulative delivery rate per aspiration IVF/ICSI cycle in POSEIDON patients: a real-world evidence study of 9073 patients. Hum Reprod. (2021) 36:2157–69. doi: 10.1093/humrep/deab152 PMC8289325 34179973

[36] Chen YH Wang Q Zhang YN Han X Li DH Zhang CL. Cumulative live birth and surplus embryo incidence after frozen-thaw cycles in PCOS: how many oocytes do we need? J Assist Reprod Genet. (2017) 34:1153–9. doi: 10.1007/s10815-017-0959-6 PMC5581780 28580513

[37] Guo W Zheng X Zheng D Yang Z Yang S Yang R. Effectiveness, flexibility and safety of switching IVF to IVM as a rescue strategy in unexpected poor ovarian response for PCOS infertility patients. J Clin Med. (2023) 12:1978. doi: 10.3390/jcm12051978 36902766 PMC10003962

[38] Xu B Zhou M Cheng M Zhang D Wu X Si C. Transvaginal ovarian drilling followed by controlled ovarian stimulation from the next day improves ovarian response for the poor responders with polycystic ovary syndrome during IVF treatment: a pilot study. Reprod Biol Endocrinol. (2020) 18:7. doi: 10.1186/s12958-019-0559-7 31980027 PMC6982383

