# Supplementary material for: Live birth rate per fresh embryo transfer and cumulative live birth rate in patients with PCOS under the POSEIDON classification: a retrospective study
Source: Front Endocrinol (Lausanne). 2024 May 28;15:1348771. doi: 10.3389/fendo.2024.1348771 (PMC11165210; doi:10.3389/fendo.2024.1348771)
Supplement: Supplementary file 1 [file Table_1.docx]

**Supplemental Table 1** **Pregnancy outcomes of women with PCOS <35 years old stratified according to the POSEIDON criteria**

|  | POSEIDON | | | | | |  | non-POSEIDON |
| --- | --- | --- | --- | --- | --- | --- | --- | --- |
|  | **1a(n=44)** | ***P* (1a)** | **1b(n=430)** | ***P* (1b)** | ***P* (1a vs. 1b)** | **1(n=474)** | ***P* 1** | **3(n=1618)** |
| ET cancel rate % (n) | 15.9(7/44) | 0.003 | 12.1(52/430) | <0.001 | 0.465 | 12.4(50/474) | <0.001 | 38.1(617/1618) |
| Type of transfer |  |  |  |  |  |  |  |  |
| Fresh | 77.3 (34/44) | <0.001 | 63.7(274/430) | <0.001 | <0.001 | 65.0(308/474) | <0.001 | 37.5(607/1618) |
| FET | 2.3(1/44) |  | 10.2(44/430) |  |  | 9.5(45/474) |  | 39.5(639/1618) |
| Fresh + FET | 6.8(3/44) |  | 24.2(104/430) |  |  | 22.6(107/474) |  | 22.4(362/1618) |
| No Transfer | 13.6(6/44) |  | 1.9(8/430) |  |  | 3.0(14/474) |  | 0.6(10/1618) |
| Fresh cycles (n=1620) | **n=37** |  | **n=378** |  |  | **n=415** |  | **n=1001** |
| Embryo transferred (n) | 2(1,2) | 0.601 | 2(2,2) | 0.102 | 0.003 | 2(2,2) | 0.682 | 2(0,2) |
| Blastocyte transferred (%) | 0 | - | 2.1(8/378) | <0.001 | - | 1.9(8/415) | <0.001 | 10.5(105/1001) |
| Implantation rate % (n) | 33.9(21/62) | 0.033 | 42.8(307/717) | <0.001 | 0.048 | 42.1(239/411) | 0.010 | 47.6(879/1847) |
| Clinical pregnancy rate % (n) | 43.2(16/37) | 0.014 | 60.1(227/378) | 0.276 | 0.038 | 58.6(243/415) | 0.105 | 63.2(633/1001) |
| LBR per transfer fresh % (n) | 37.8 (14/37) | 0.035 | 50.0(189/378) | 0.963 | 0.158 | 48.9(203/415) | 0.026 | 55.4(555/1001) |
| FET cycles (n=1898) | **n=4** |  | **n=188** |  |  | **n=192** |  | **n=1490** |
| LBR per transfer FET % (n) | 50.0(2/4) | 0.556 | 33.5(63/188) | <0.001 | 0.490 | 33.9(65/192) | <0.001 | 50.8 (757/1490) |
| CLBR % (n) | 36.4(16/44) | <0.001 | 58.6(252/430) | <0.001 | 0.005 | 56.5(268/474) | <0.001 | 81.0(1310/1618) |
| Conception mode % (n) |  |  |  |  |  |  |  |  |
| IVF/ICSI fresh % (n) | 87.5(14/16) | <0.001 | 75.0(189/252) | <0.001 | 0.258 | 75.7(203/268) | <0.001 | 42.4(558/1310) |
| IVF/ICSI FET % (n) | 12.5(2/16) |  | 25.0 (63/252) |  |  | 24.3(65/268) |  | 57.6(755/1310) |

CLBR, cumulative live birth rate: cumulative delivery rate from one aspiration IVF/ICSI cycle; *P (1a)*, *P*-value between Group 1a and Group 3; *P (1b)*, *P*-value between Group 1b and Group 3; *P (1a vs. 1b), P*-value between Group 1a and Group 1b; *P1*, *P*-value between Group 1 (combined 1a+1b) and Group 3.

**Supplemental Table 2 Pregnancy outcomes of women with PCOS ≥35 years old stratified according to the POSEIDON criteria**

|  | **POSEIDON** | | | | | |  | **non-POSEIDON** |
| --- | --- | --- | --- | --- | --- | --- | --- | --- |
|  | **2a(n=6)** | ***P* (2a)** | **2b(n=67)** | ***P* (2b)** | ***P* (2a vs. 2b)** | **2(n=73)** | ***P (*2)** | **4(n=212)** |
| **ET cancel rate % (n)** | 33.3(2/6) | 1.000 | 4.5(3/67) | <0.001 | 0.066 | 6.7(5/75) | <0.001 | 35.8(76/212) |
| **Type of transfer** |  |  |  |  |  |  |  |  |
| Fresh | 50(3/6) | 0.004 | 77.6(52/67) | <0.001 | 0.096 | 75.3(55/73) | <0.001 | 42.0(89/212) |
| FET | 0 |  | 3.0(2/67) |  |  | 2.7(2/73) |  | 34.9(74/212) |
| Fresh + FET | 16.7(1/6) |  | 17.9(12/67) |  |  | 17.8(13/73) |  | 22.2(47/212) |
| No Transfer | 33.3(2/6) |  | 1.5(1/67) |  |  | 4.1(3/73) |  | 0.9(2/212) |
| **Fresh cycles (n=1620)** | **n=4** |  | **n=64** |  |  | **n=68** |  | **n=136** |
| Embryo transferred (n) | 1(0,1.3) | 0.340 | 2(2,3) | 0.002 | 0.001 | 2(2,2) | 0.242 | 2(0,2) |
| Blastocyte transferred (%) | 0 | - | 6.3(4/64) | 0.067 | - | 5.9(4/68) | 0.068 | 15.4(21/136) |
| Implantation rate % (n) | 20.0(1/5) | 0.366 | 37.3(53/142) | 0.551 | 0.430 | 36.7(54/147) | 0.181 | 43.7(124/284) |
| Clinical pregnancy rate % (n) | 25.0(1/4) | 0.214 | 62.5 (40/64) | 0.474 | 0.137 | 60.3(41/68) | 0.350 | 67.6(92/136) |
| LBR per transfer fresh % (n) | 25.0(1/4) | 0.439 | 51.6(33/64) | 0.442 | 0.303 | 40.0(34/68) | 0.371 | 57.4(78/136) |
| **FET cycles (n=1898)** | **n=1** |  | **n=15** |  |  | **n=16** |  | **n=200** |
| LBR per transfer FET % (n) | 100.0(1/1) | 0.835 | 13.3(2/15) | 0.044 | 0.188 | 20.0(3/15) | 0.115 | 39.5(79/200) |
| **CLBR % (n)** | 33.3(2/6) | 0.080 | 52.2(35/67) | 0.001 | 0.645 | 50.7(37/73) | <0.001 | 74.1(157/212) |
| Conception mode % (n) |  |  |  |  |  |  |  |  |
| IVF/ICSI fresh % (n) | 50(1/2) | 1.000 | 94.3(33/35) | <0.001 | 0.099 | 91.9(34/37) | <0.001 | 49.7(78/157) |
| IVF/ICSI FET % (n) | 50(1/2) |  | 5.7(2/35) |  |  | 8.1(3/37) |  | 50.3(79/157) |

CLBR, cumulative live birth rate: cumulative delivery rate from one aspiration IVF/ICSI cycle; *P (2a)*, *P*-value between Group 2a and Group 4; *P (2b)*, *P*-value between Group 2b and Group 4; *P (2a vs. 2b), P*-value between Group 2a and Group 2b*; P2*, *P*-value between Group 2 (combined 2a+2b) and Group 4.
