# Supplementary figures and images for: Live birth rate per fresh embryo transfer and cumulative live birth rate in patients with PCOS under the POSEIDON classification: a retrospective study
Source: Front Endocrinol (Lausanne). 2024 May 28;15:1348771. doi: 10.3389/fendo.2024.1348771 (PMC11165210; doi:10.3389/fendo.2024.1348771)

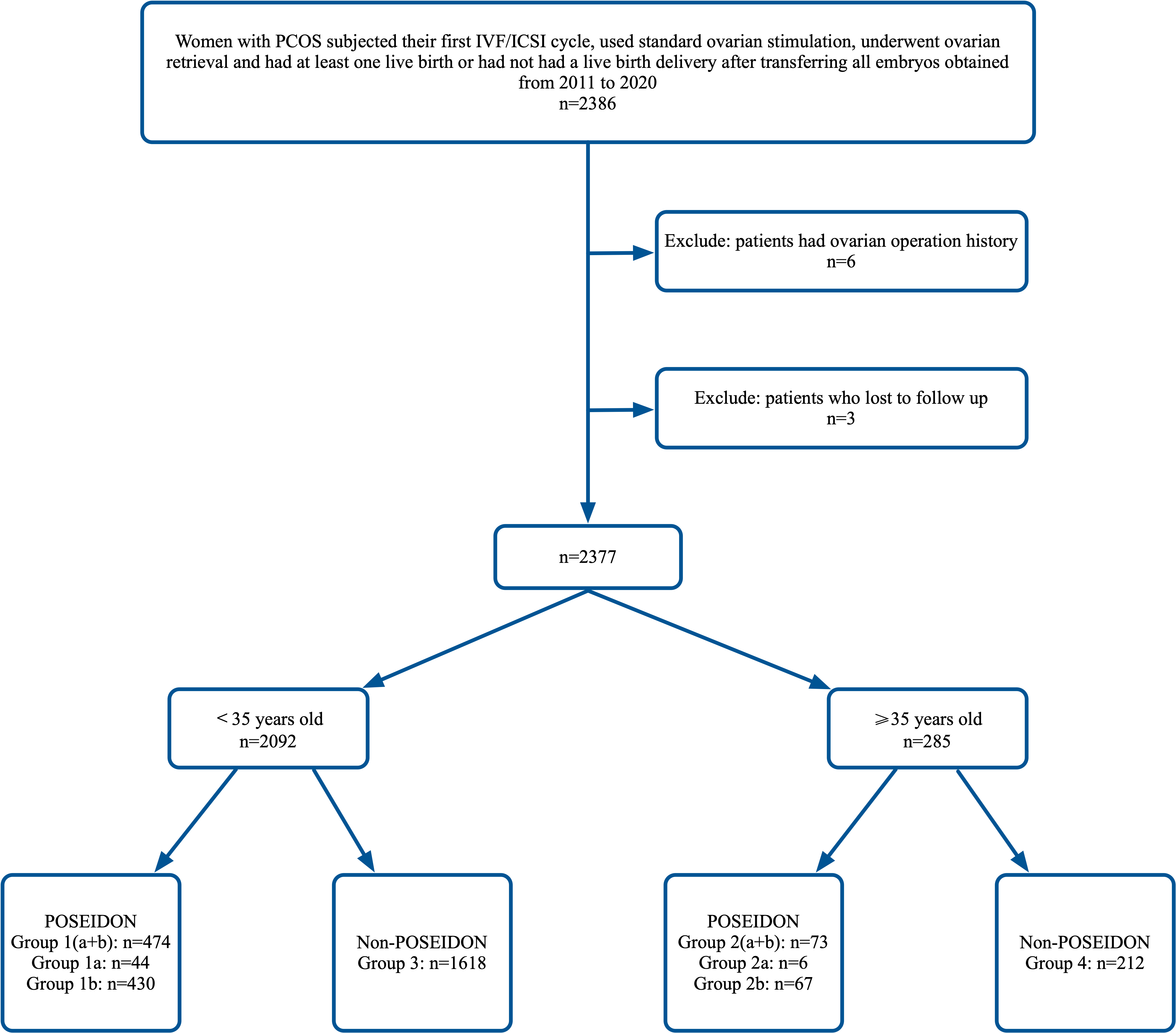

Supplement: Supplementary Figure 1 — Flow diagram showing total patient breakdown. POSEIDON Group 1 (Group 1): Age <35 years and AFC ≥5. Subgroup 1a included patients with fewer than four oocytes retrieved, and Subgroup 1b included patients with four to nine oocytes retrieved. POSEIDON Group 2 (Group 2): Age ≥35 years and AFC ≥5. Subgroup 2a included patients with fewer than four oocytes retrieved, and Subgroup 2b included patients with four to nine oocytes retrieved. Non-POSEIDON (Group 3 and Group 4): Patients with AFC ≥5 and >9 oocytes retrieved. Group 3 included patients aged <35 years, and Group 4 included patients aged 35 years and older. [file Image_1.jpeg]

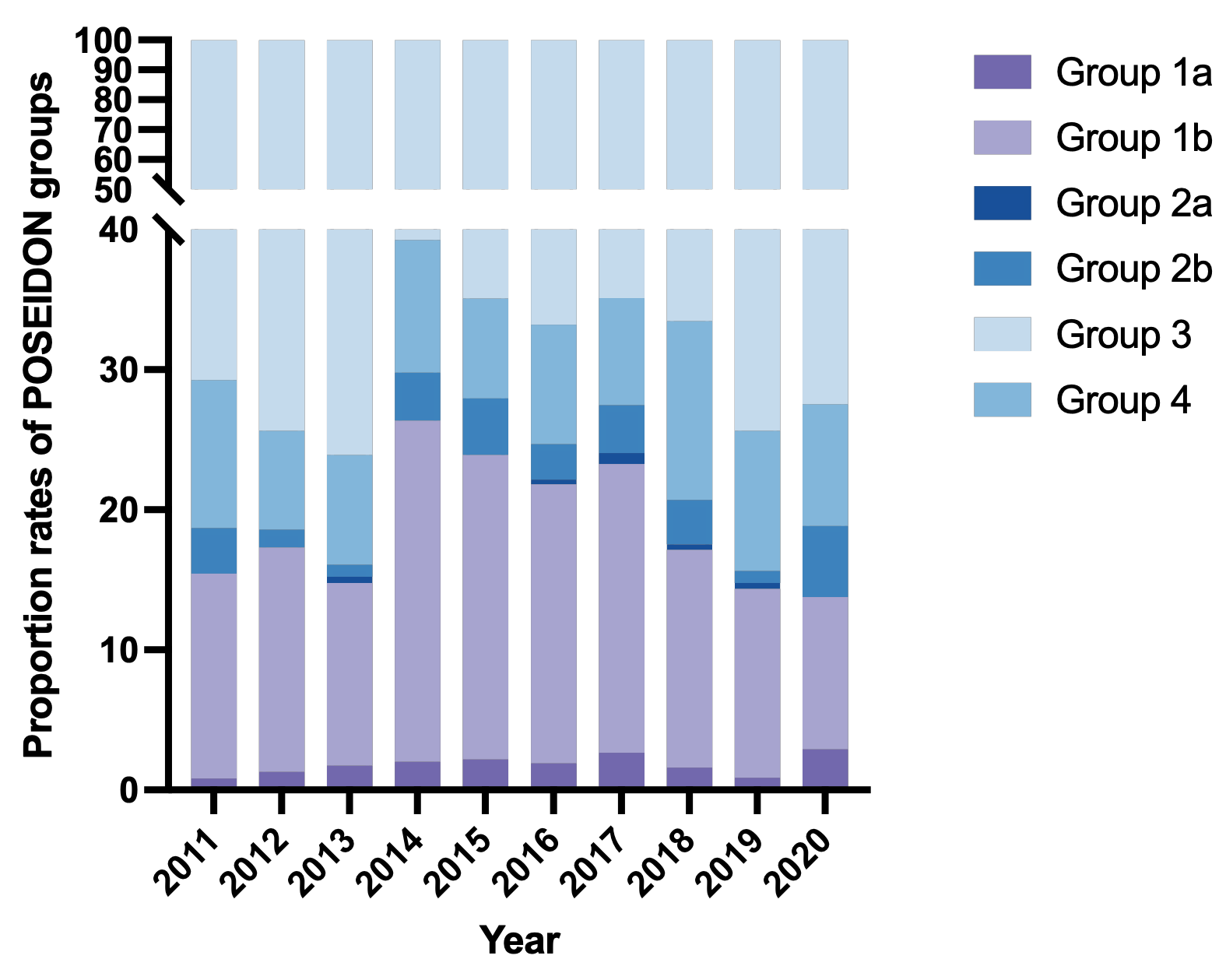

Supplement: Supplementary Figure 2 — Bar chart showing the distribution of patients in each POSEIDON group in different years from 2011 to 2020. [file Image_2.tiff]
